# Biomimetic doxorubicin/ginsenoside co-loading nanosystem for chemoimmunotherapy of acute myeloid leukemia

**DOI:** 10.1186/s12951-022-01491-w

**Published:** 2022-06-14

**Authors:** Mo Chen, Yingyu Qiao, Jie Cao, La Ta, Tianyuan Ci, Xue Ke

**Affiliations:** 1 Department of Pharmaceutics, China Pharmaceutical University, Xuanwumen Campus, No.24, Tongjiaxiang, Gulou District, Nanjing, 210009 Jiangsu province China; 2 Department of Pharmaceutical Sciences, Shanghai University of Traditional Chinese Medicine, Shanghai, 201203 China

**Keywords:** Acute myeloid leukemia, Chemoimmunotherapy, Ginsenoside, Leukemia cells targeting

## Abstract

**Background:**

Acute myeloid leukemia (AML) showed limited clinical therapeutic efficiency with chemotherapy for its multi-distributed lesions and hard-to-kill leukemia cells deep in the bone marrow.

**Results:**

Here, a biomimetic nanosystem (DR@PLip) based on platelet membrane (PM) coating and doxorubicin (DOX)/ginsenoside (Rg3) co-loading was developed to potentiate the local-to-systemic chemoimmunotherapy for AML. The PM was designed for long-term circulation and better leukemia cells targeting. The participation of Rg3 was proved to enhance the tumor sensitivity to DOX, thus initiating the anti-tumor immune activation and effectively combating the leukemia cells hiding in the bone marrow.

**Conclusions:**

In conclusion, the strategy that combining immediate chemotherapy with long-term immunotherapy achieved improved therapeutic efficiency and prolonged survival, which provided a new perspective for the clinical treatment of AML.

**Supplementary Information:**

The online version contains supplementary material available at 10.1186/s12951-022-01491-w.

## Introduction

Acute myeloid leukemia (AML) is a hematological malignant tumor originating from hematopoietic cells. It is mainly manifested by the malignant proliferation, blocked differentiation, and disordered apoptosis of myeloid cells [1–3], hence destroying the normal hematopoietic function. The poor prognosis of AML contributes to a five-year survival rate of only 30% [1, 4, 5]. Even though hematopoietic cell transplantation (HSCT) could realize disease regression [6–8], the difficulties in searching matching cells [8, 9] as well as transportation-related mortality sometimes limit the application to all patients, and chemotherapy is still the most conventional treatment in AML therapy. However, the leukemia cells in bone marrow are sometimes hard to kill due to the poor distribution of therapeutics and the patho-microenvironment of bone marrow that inactivate the cytotoxic effects of the chem-drugs, which leads to a high recurrence rate [10–12]. Thus, the development of a strategy that could target the leukemia cells and sensitive these tumor cells to chemotherapy is of significance.

Immune cells have the natural advantage of infiltrating deep tissues to attack tumors, which provides favorable conditions for eradicating leukemia cells in the bone marrow [13]. Ginsenoside (Rg3) is a natural anti-tumor drug derived from ginseng [14]. In particular, the combination of Rg3 in chemotherapy was proved to enhance the tumor sensitivity to chemotherapeutic drugs. The improved killing efficiency could facilitate the full exposure of tumor antigens, thereby enhancing the anti-tumor immune response rate [15, 16].

**Fig. 1:**
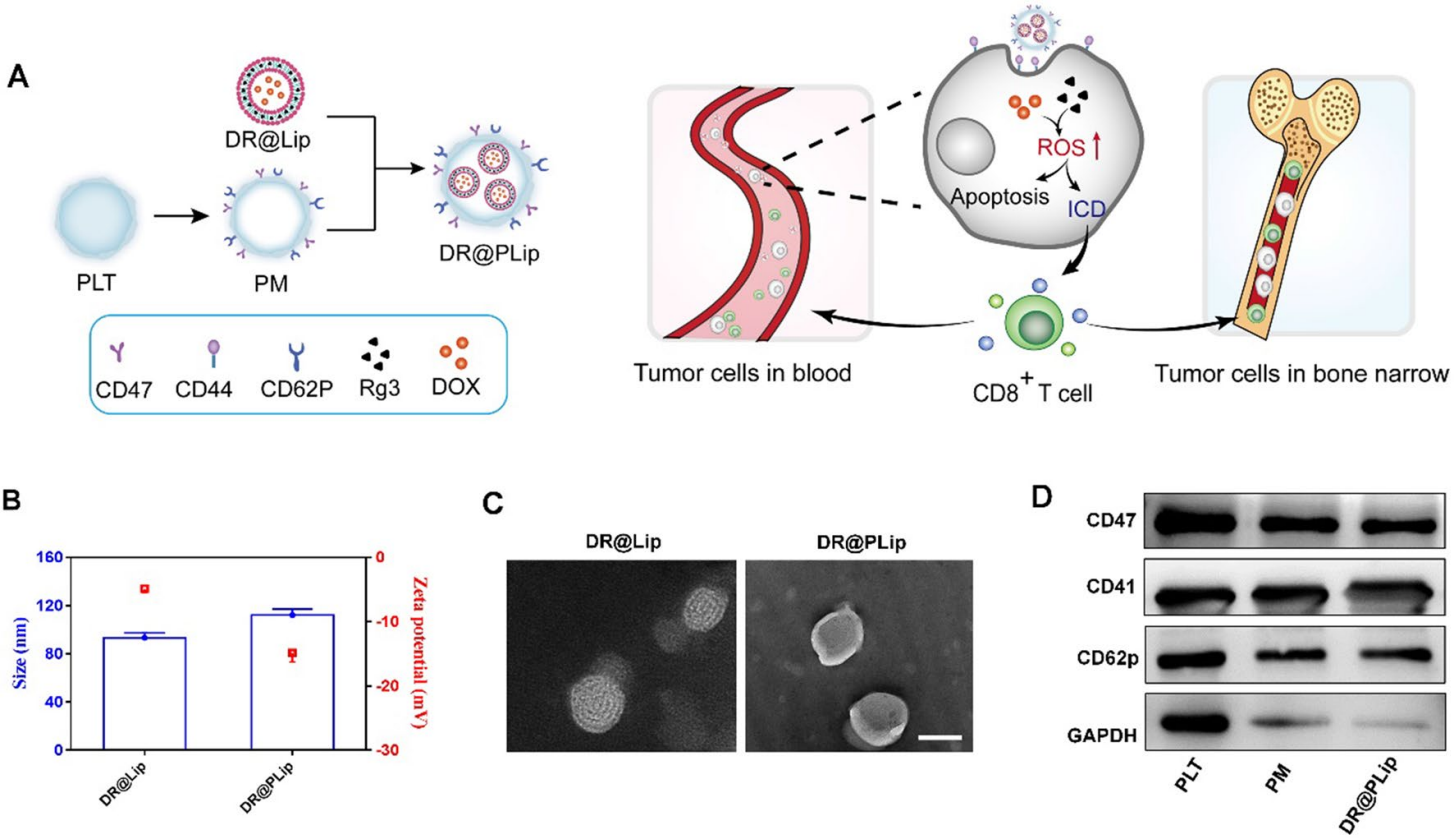
Characteristic and anti-tumor mechanism of the biomimetic nanosystem (DR@PLip). **A** Preparation and anti-tumor mechanism of DR@PLip. Lipsomes co-loading DOX and Rg3 were co-extruded with PM to construct the DR@PLip. The biomimetic nanosystem in blood encountered the AML cells and induced powerful immunogenic cell death. Then the activated effector T cells penetrated deep in bone marrow to kill the AML cells, as well as eliminated the AML cells remained in blood. **B** Size distribution and zeta potential of the nanosystem. **C** TEM images of the nanosystem. Scale bar, 100 nm. **D** The PLT relative protein detected in different samples

Here, we constructed PM coated DOX/Rg3 liposomes as a biomimetic nanosystem (DR@PLip) for the treatment of AML (Fig. 1A). The "don't eat me" signal associated with CD47 on the PM prevented the nanosystem from being cleared by the endothelial reticulum system [17, 18]. In addition, the PM benefited recognization and binding of the nanosystem to AML cells by the P-selectin (CD62p)/CD44 interaction, which was conducive to the targeted attack of the AML cells [19–21]. Moreover, Rg3 as a reactive oxygen species (ROS) inducer was synergized with DOX to facilitate sufficient tumor antigen exposure and boost immune response [22]. The activated T cells combined with PD-L1 antibody (aPD-L1) could track and combat the fatal residual lesions hiding in the bone marrow [13, 23], achieving long-term supervision of AML recurrence [24, 25].

## Materials and methods

### Materials, cells and animals

Egg phospholipids (EPC) was purchased from m A.V.T. Pharmaceutical Co., Ltd. (Shanghai, China). Doxorubicin Hydrochloride (DOX.HCl) and Cholesterol was brought from Aladdin Reagent Co., Ltd. (Shanghai, China). Ginsenoside (Rg3) was purchased from Nanjing Plant Origin Biological Technology Co., Ltd. (Nanjing, China). DMEM medium and MTT were purchased from KeyGen biotech (Nanjing, China). PD-L1 antibody (aPD-L1, B7-H1) was brought from BioXcell Co., Ltd. (Shanghai, China). The Annexin V-FITC/PI Apoptosis Detection Kit (Cat No.KGA107) and collagenase was bought from KeyGen biotech (Nanjing, China). Anti-Firefly Luciferase antibody [EPR17790] was brought from abcam. Anti-CD11c, anti-CD80, anti-CD86, anti-CD3, anti-CD4, anti-CD8, anti-CD44, anti-CD62L and interferon-*γ* (IFN-*γ*) and tumor necrosis factor-*α* (TNF-*α*) enzyme-linked immunosorbent assay (ELISA) kits (Cat: No 430804, Cat: No 430904) were brought from Biolegend (USA). C1498 cells, Luciferase-expressing C1498 cells (C1498-luc cells) and RAW264.7 cells were cultured in DMEM cell culture medium with 10% fetal bovine serum (FBS) and 1% penicillin/streptomycin at 37 ºC in a 5% CO_2_ atmosphere. C57 mice (female, 18–22 g) were supplied by GemPharmatech LLC Co., Ltd. (Nanjing, China). The animals involved in this study were treated according to protocols evaluated and approved by the ethical committee of China Pharmaceutical University.

### Extraction of PM

Whole blood from C57BL/6 mice was mixed with 4% sodium citrate solution at volume ratio of 9:1. Then the mixture was centrifuged at 200*g* for 20 min to separate most plasma. Then centrifugation was performed again (200*g*, 20 min) to collect the upper plasma and white platelets (PLTs) at the interface between the plasma and red blood cells. Then the collection was centrifuge at 800*g* for 15 min to obtain PLTs. The PLTs were resuspended in 1 ml of PBS (pH 7.4) and then performed freeze–thaw cycles (-80 °C/25 °C) for three times. Finally, centrifugation (12,000 rpm, 10 min) was performed to obtain the platelet membrane (PM).

### Preparation and characterization of DR@Lip and DR@PLip

About 12 mg of DOX.HCl was dissolved by 1.2 ml of DMSO and 10 μl of triethylamine, and stirred for 24 h in the dark to prepare a desalted DOX solution. Then the solution was dialyzed (MWCO 1 kDa) and lyophilized to obtain desalted DOX. D@Lip or DR@Lip were prepared as the film dispersion method. Briefly, EPC, cholesterol or Rg3, desalted DOX at the ratio of 16:3:1 (w/w/w) were dissolved in tetrahydrofuran (THF) in a round bottom flask. The THF was removed by rotary evaporation at 37 °C, forming a uniform lipid film. Then distilled water was added to hydrate at 37 °C for 1 h. Finally, the sonication was performed to obtain D@Lip or DR@Lip.

DR@Lip and PM were mixed and co-extruded through an AVANTI mini-extruder (100 nm polycarbonate porous membrane, AVT-USA) to prepare DR@PLip. The particle size and zeta potential of the liposomes were detected by dynamic light scattering (DLS) spectrophotometer (Brookhaven Instruments, USA). The morphology of DR@Lip and DR@PLip were observed by transmission electron microscope (TEM). UV spectrophotometer was used to detect the drug loading and encapsulation efficiency of DOX in liposomes.

Proteins on DR@PLip were detected by western blotting as the same method described before [26, 27]. Briefly, the proteins extracted from PLT, PM and DR@PLip were analyzed by BCA assay kit, and the proteins of each group were adjusted to be consistent, mixed with SDS-PAGE loading buffer and heated at 100 °C for 20 min. For western blotting experiments, proteins were separated by 12% SDS–polyacrylamide gel electrophoresis (SDS-PAGE) and transferred using 0.45 μm PVDF membranes (Millipore). After blocking with 5% BSA, PVDF membranes were incubated with antibodies against cell membrane markers (CD47, CD41, CD62p, GAPDH) overnight at 4 °C. After further incubation with secondary antibody for 2 h. Membranes were imaged with ECL western blotting substrate (Thermo Scientific, USA) and developed with a Chemidoc imaging system (Bio-Rad).

The stability of DR@PLip was also evaluated by using DLS spectrophotometer. The particle size and polydispersity index of DR@PLip were detected at certain time points (0 h, 2 h, 4 h, 8 h, 12 h, 24 h, and 48 h) in DMEM medium + 10% FBS.

For the drug release assay, free DOX and DR@PLip in dialysis bag (MWCO 1000 Da) were immersed in PBS (pH 7.4) for 48 h respectively, and the external solution was collected at predetermined timepoints. The released DOX was determined by the fluorescent spectrophotometry with excitation and emission wavelength of 494 nm and 554 nm. The Rg3 released from DR@PLip was determined by the HPLC (Shimadzu, LC-2010).

### Cell uptake

Flow cytometry and laser confocal scanning microscope (CLSM) were used to evaluate the cell uptake of different preparations. Briefly, C1498 cells were planted in confocal dishes and incubated at 37 °C for 24 h. DR@Lip and DR@PLip with the same DOX concentration was added and incubated for 4 h. Then the uptake of DR@Lip and DR@PLip by C1498 cells was observed by CLSM (Carl Zeiss LSM 700). In addition, the C1498 cells were collected and the uptake effect was detected by flow cytometry (BD FACS Celesta). The same method was used to evaluate the uptake of different preparations by RAW264.7 cells.

### Cytotoxicity

C1498 cells in the logarithmic growth phase were planted in a 96-well plate (5 × 10^3^/well), and incubated at 37 °C for 24 h. Then cells were treated with different preparations (DOX 2.5 μg/ml, Rg3 7.5 μg/ml) for 24 h. Then 15 μl of MTT (15 mg/ml) was added to each well and incubated for another 4 h. Finally, the supernatant was aspirated, 150 μl of DMSO was added and incubated at 37 °C for 30 min. The absorbance at 490 nm was measured with a microplate reader. In order to evaluate the cell apoptosis effect induced by the preparations, cells collected were stained with Annexin V and PI and detected by flow cytometry (BD FACS Celesta).

### ROS production

A Fluorescent probe called Dichlorofluorescin diacetate (DCFH-DA) was used to detect the ROS content of C1498 cells after different treatments. C1498 cells were planted in a 12-well plate (1 × 10^5^/well) and incubated at 37 °C for 24 h. Then cells were treated with different preparations (DOX 1 μg/ml, Rg3 3 μg/ml) for 12 h. Then DCFH-DA (5 μM) was added and incubated for 30 min. Finally, the intracellular ROS content was quantitatively detected by flow cytometry (BD FACS Celesta).

### ICD and DC maturation in vitro

Tumor immunogenic death (ICD) including calreticulin (CRT) exposure, high mobility histone B1 (HMGB1) and adenosine triphosphate (ATP) release of tumor cells. Flow cytometry was used to detect the expression of CRT exposure on C1498 cells. Briefly, C1498 cells were seeded in a 12-well plate (1 × 10^5^/well) and incubated at 37 °C for 24 h. Then cells were treated with different preparations (DOX 1 μg/ml, Rg3 3 μg/ml) for 12 h. Then CRT-labeled antibody was added and incubated at 4 °C for 1 h. Afterwards, CRT exposure of the collected cells was detected by flow cytometry (BD FACS Celesta). In addition, the content of HMGB1 and ATP in the supernatant were determined by ELISA kit and ATP detection kit respectively.

The bone marrow dendritic cells (BMDCs) were extracted from C57BL/6 mice with the same method described before [28]. Briefly, white cells collected from bone marrow were seeded into a six-well plate (1 × 10^6^/well), then 5 ng/ml interleukin-4 (IL-4) and 20 ng/ml Granulocyte–macrophage Colony Stimulating Factor (GM-CSF) were added to induce differentiation into dendritic cells (DCs). On day 7, C1498 cells treated with different preparations and supernatant were incubated with DCs. Cells were collected on day 9 and resuspended in anti-CD11c FITC, anti-CD80 PE, anti-CD86 APC containing staining buffer. The expression of CD80 and CD86 of DCs were detected by flow cytometry (BD FACS Celesta) to evaluate the DC maturation induced by cells under different treatments.

### Pharmacokinetics

Eight SD rats (180–200 g) were randomly divided into 2 groups (*n* = 4). Then rats were injected with DR@Lip or DR@PLip at a dose of 5 mg/kg through the tail vein. Blood samples were taken from the orbit at 1, 2, 4, 6, 8, 12, 24, 36, and 48 h after injection and centrifuged at 10,000 rpm for 10 min to collect the upper plasma. About 200 μl of plasma was mixed with 3800 μl of hydrochloric acid (0.3 M)/ethanol mixture (3:7, v/v) and placed at −20℃ for 48 h. Then samples were centrifuged at 10,000 rpm for 10 min to collect the plasma. Finally, the DOX fluorescence intensity of each sample was detected by fluorescence spectrophotometer, The methodology was established and validated with the same method described before [29], and the main pharmacokinetic parameters was calculated by using winnonlin 6.3 software.

### Anti-tumor efficacy in vivo

In order to evaluate the anti-tumor effect of different preparations in vivo, C1498-luc cells (5 × 10^6^/mouse) were inoculated into C57BL/6 mice through the tail vein to construct an AML model [28]. On day 7, the mice were randomly divided into 5 groups (*n* = 9) and given different treatments: saline, D@Lip, DR@Lip, DR@PLip and DR@PLip + aPD-L1 (DOX 5.0 mg/kg, aPD-L1 2.5 mg/kg). Preparations were administered via the tail vein on day 8, 11, 14, and 17. The bioluminescence imaging of mice were captured on day 7, day 14 and day 21. The survival of mice remained in different groups were monitored to 60 days.

On day 22, three mice of each group were sacrificed to collect cells from bone marrow. After treated with ack buffer, the samples were conducted fixation and permeabilization, and stained with anti-firefly luciferase antibody and Cy5 conjugated goat anti-rabbit IgG. Then, the proportion of C1498-luc cell in bone marrow was detected by flow cytometry (BD FACS Verse).

### Immune activation in vivo

In order to evaluate the immune activation effect of different preparations, three C1498-luc cells bearing mice in each group were sacrificed on day 13, and the lymph nodes were extracted and treated with the same method as described before [30]. Briefly, lymph nodes were digested in collagenase containing (0.05 mg/ml) DMEM medium, filtrated through a cellular filter (100 μm), washed with PBS and centrifuged (500 g, 5 min). Then the cells were stained with anti-CD11c FITC, anti-CD80 APC and anti-CD86 PE antibodies, and the DC maturation was detected by a flow cytometer (BD FACS Verse). On day 13, 200 μl of blood sample was taken from the mouse orbit. After treating with Ammonium Chloride Potassium lysis buffer (ACK buffer) for red blood cell lysis, it was stained with anti-CD3 FITC, anti-CD4 PE and anti-CD8 APC antibodies, and the CD8/CD4 T cells were detected by flow cytometry (BD FACS Verse). Meanwhile, the tumor necrosis factor-*α* (TNF-*α*) and interferon-*γ* (IFN-*γ*) in the serum were detected by ELISA kit. On day 22, the mice were sacrificed, the peripheral monocytes from their hind leg bones were collected. After treating with ACK buffer, the samples were stained with anti-CD3 FITC, anti-CD4 PE and anti-CD8 APC antibodies, and the CD8/CD4 T cells were detected by flow cytometry (BD FACS Verse).

The proportion of effector memory T cells (T_EM_ cells) in spleens of different groups was also detected on day 22. The collected lymphocytes were stained with anti-CD3 FITC, anti-CD8 Percp-Cy5.5, anti-CD44 APC and anti-CD62L PE antibodies. Then the T_EM_ cells were detected by flow cytometry (BD FACS Verse).

### Biosafety evaluation in vivo

Healthy C57 mice were randomly divided into two groups (*n* = 3) and treated with saline or DR@PLip + aPDL1 (DOX 5.0 mg/kg, aPD-L1 2.5 mg/kg) for 4 times. Then major organs (heart, liver, spleen, lung and kidney) were collected for H&E staining analysis (Wuhan Servicebio Technology).

### Statistical analysis

All data were representative results of at least three independent experiments. The results were expressed as mean values ± SD. The data were analyzed by Student’s t-test between two groups and ordinary one-way ANOVA for three or more groups. The survival curves were analyzed via the log-rank (Mantel-Cox) test. All statistical analysis was conducted by the GraphPad Prism Software.

## Results and discussions

### Preparation and characterization of DR@Lip and DR@PLip

DR@Lip was prepared by the thin-film dispersion method. The hydrodynamic particle size and the zeta potential of DR@Lip were about 100 nm and −4.83 mV, DR@PLip was prepared by co-extrusion of DR@Lip and PM, with increased particle size and decreased zeta potential (115 nm and −14.9 mv) (Fig. 1B). Through TEM observation (Fig. 1C), obvious double-layer structure was found on the outermost layer of DR@Lip and DR@PLip. As shown in Fig. 1D, the expression of related membrane characteristic proteins of PLT and DR@PLip were consistent, further indicating the successful construction of biomimetic system. The drug loading of DOX measured by UV spectrophotometer was 4.93%, and the encapsulation efficiency was 82.92%. DR@PLip also showed desirable plasma stability, which is beneficial to in vivo application (Additional file 1: Figure S1). As shown in, Additional file 1: Figure S3, the in vitro DOX release of DR@PLip was slower than that of free DOX. Besides, DR@PLip displayed a sustained release of Rg3, and the cumulative release percentage reached 30% within 25 h (Additional file 1: Figure S4).

### Cell uptake

**Fig. 2:**
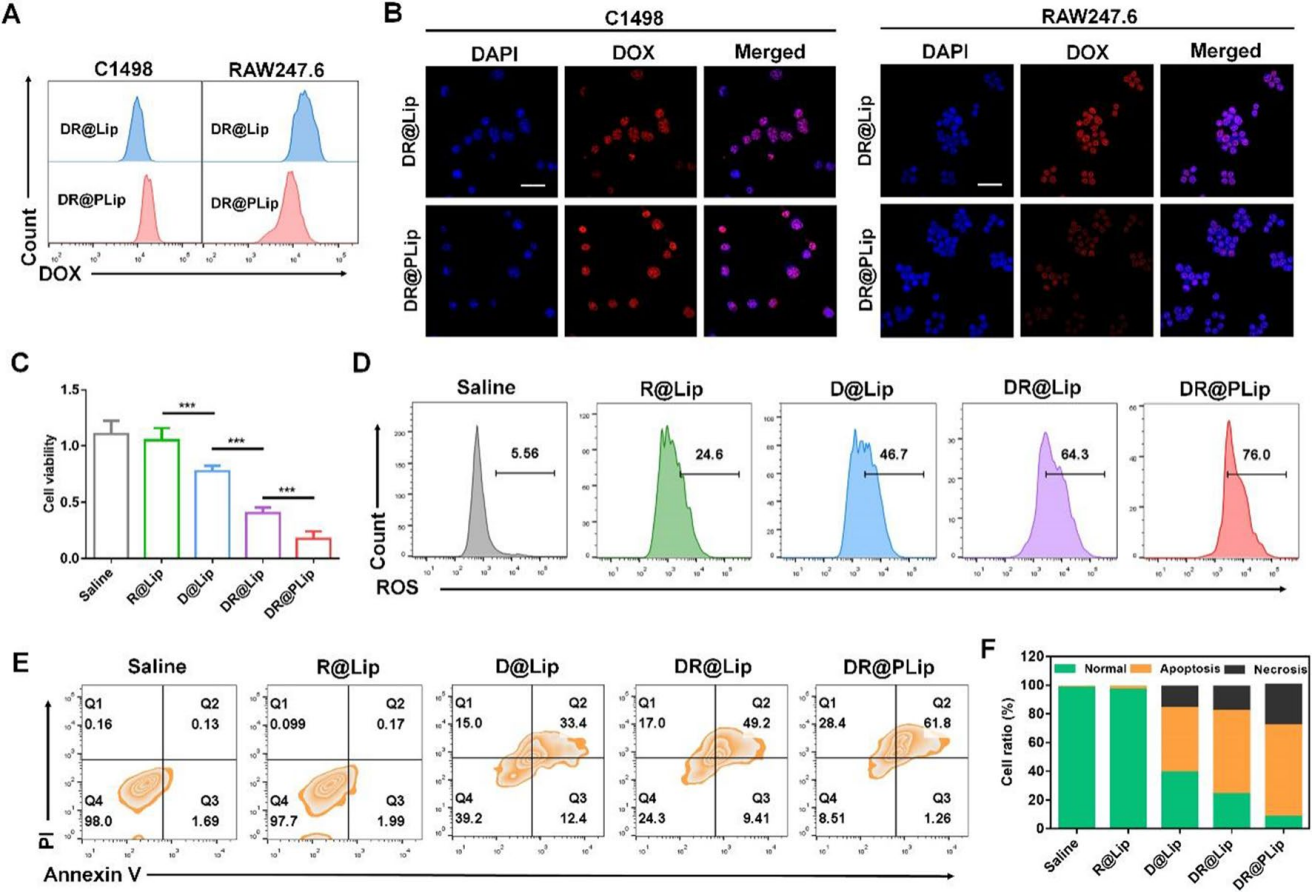
Cell viability in vitro. Cellular uptake of the nanosystem by C1498 cells and RAW247.6 cells analyzed by (**A**) flow cytometry and (**B**) CLSM. Scale bar, 40 μm. **C** Cytotoxicity of different treatments (*n* = 3). **D** ROS generation in C1498 cells with different treatments. **E** Apoptosis assay of C1498 cells with different treatments. **F** Statistical analysis of (**E**). Data are shown as mean ± SD. *P* values were calculated by Student's t-test, ***p < 0.001

Selective cell uptake helps improve treatment efficiency. On one hand, increased uptake of the preparation by C1498 cells is necessary for cytotoxicity. As shown in Fig. 2A & B and Additional file 1: Figure S2, the uptake of DR@PLip by C1498 cells was more than that of DR@Lip at 4 h, which was benefited from the CD44/CD62p interaction [18]. On the other hand, decreased uptake of the preparation by RAW264.7 cells is beneficial for the long circulation. It was shown that at 4 h, the uptake of DR@PLip by RAW264.7 cells was less than that of DR@Lip, thanks to the "don't eat me" signal of PM. Therefore, the PM coating could improve the treatment efficiency by promoting the uptake of nanosystem by tumor cells but escaping the capture by macrophages.

### Cytotoxicity

Efficient cytotoxicity is not only conducive to killing of tumor cells, but also contributes to sufficient antigen exposure, providing conditions for further immune activation. As shown in Fig. 2C, the DR@Lip group showed significantly stronger inhibition on cell viability than the D@Lip group, while the R@Lip group had almost no toxicity (Additional file 1: Figure S5). It was speculated that the increase of intracellular ROS induced by Rg3 enhanced cells sensitivity to DOX. In order to further explore its synergistic mechanism, DCFH-DA was used to detect the produce of intracellular ROS after different treatment. As shown in Fig. 2D, cells treated with R@Lip showed an enhanced fluorescence signal compared with the saline group, which was attributed to the ROS inducer effect of Rg3. Of note, cells treated with DR@Lip showed increased ROS production than D@Lip, which was inferred that Rg3 had a synergistic effect on DOX. At last, DR@PLip showed the strongest fluorescence signal, which may be in part associated with the CD44/CD62p interaction. Annexin V-FITC/propidium iodide double staining Kit was further used to evaluate the cell apoptosis. As shown in Fig. 2E & F, the DR@PLip group had strongest apoptosis effect than the other groups due to the enhanced cell uptake and promoted cytotoxicity.

### ICD and DC maturation in vitro

**Fig. 3:**
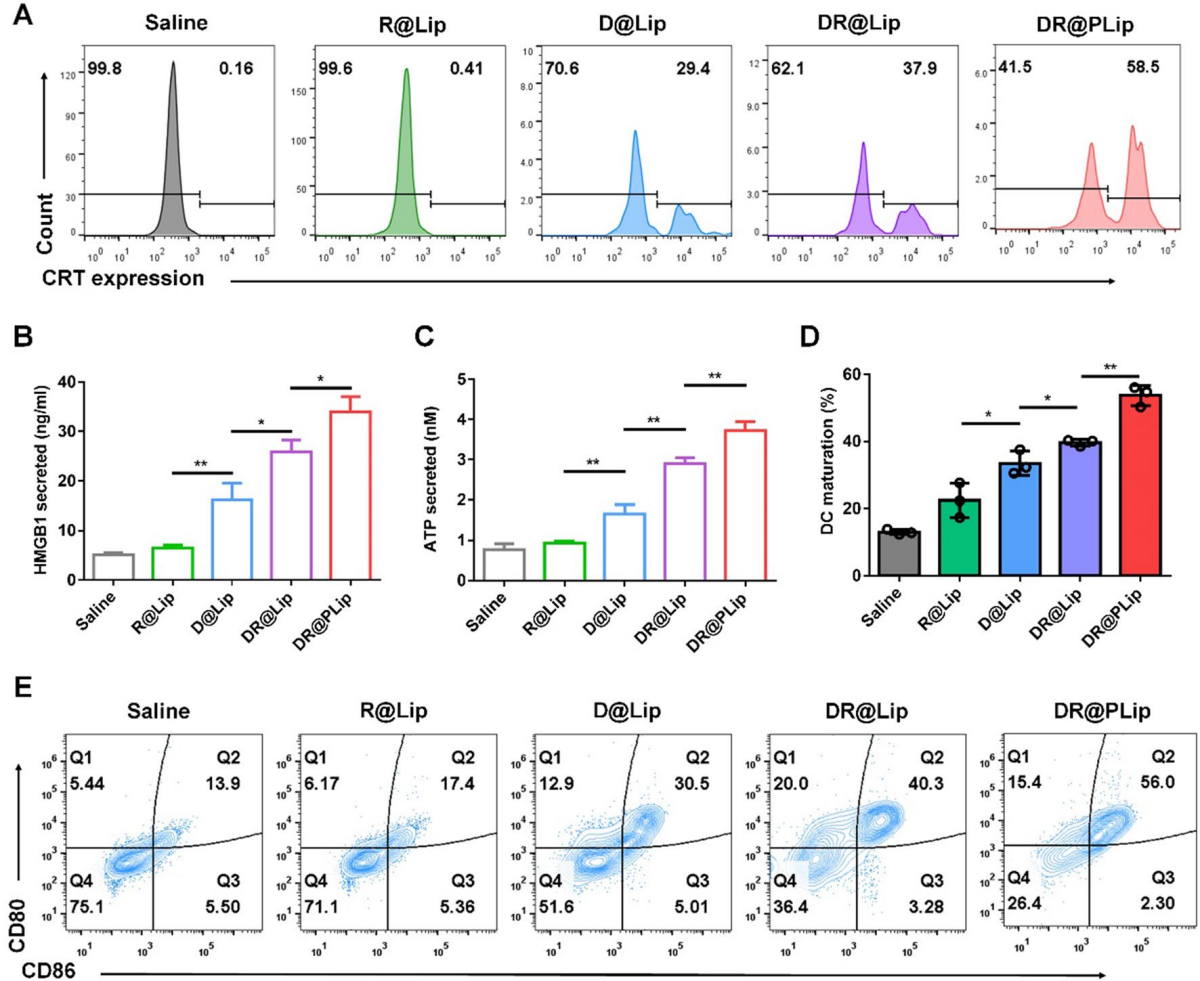
Immuno-activation in vitro. **A** CRT exposure in C1498 cells with different treatments analyzed by flow cytometry. **B** High-mobility group protein B1 (HMGB1) detected by enzyme-linked immunosorbent assay (ELISA) (*n* = 3). **C** Adenosine triphosphate (ATP) detected by the enhanced ATP assay kit (*n* = 3). **D** Statistical analysis of (**E**). **E** DC maturation (CD80^+^CD86^+^ of CD11c^+^ DCs) after cocultured with C1498 cells with different treatments. Data are shown as mean ± SD. *P* values were calculated by Student’s t-test, *p < 0.05, **p < 0.01

It is well known that DCs are one of the most powerful antigen presenting cells (APCs) in the immune system [30]. Chemotherapy is proved to induce ICD, promoting DC maturation [31]. Mature DCs effectively processes antigens and then presents antigens to T cells, promoting the T cells activation. CRT exposure, HMGB1 and ATP release are the three most important signals in ICD [32]. As shown in Fig. 3A, the expression of CRT in the saline group and the R@Lip group were limited, while that of the D@Lip and DR@Lip groups both increased to 29.4% and 37.9%, respectively. Furthermore, the CRT exposure in the DR@PLip group (58.5%) was significantly more than other groups due to more cell uptake [33] and promoted cell toxicity. Consistently, the HMGB1 and ATP released in the DR@PLip group were also higher than other groups, indicating the powerful ICD inducing effect of DR@PLip (Fig. 3B & C) based on enhanced ROS production [34].

The mature DCs (CD80^+^ and CD86^+^) was further evaluated by flow cytometry. It was observed that the saline and R@Lip treatment had almost no effect on DC maturation, while the D@Lip, DR@Lip and DR@PLip treatment induced (30.5%, 40.3% and 56.0%) of DC maturation, respectively, which was consistent with the ICD detection (Fig. 3D& E).

### Pharmacokinetics

Benefit to the “don’t eat me” signal expressed by CD47 on the PM, the PM-coated nanoparticles could escape the phagocytosis of the reticuloendothelial system, resulting in long-lasting blood circulation time than ordinary nanoparticles [35], as shown in Additional file 1: Figure S3. Compared with DR@Lip, DR@PLip significantly enhanced the area under the distribution curve (AUC_0-t_), and DR@PLip also exhibited longer T_1/2_, which indicated the extended half-life of the biomimetic nanosystem (Additional file 1: Figure S6, Table S1).

### Anti-tumor efficacy in vivo

**Fig. 4:**
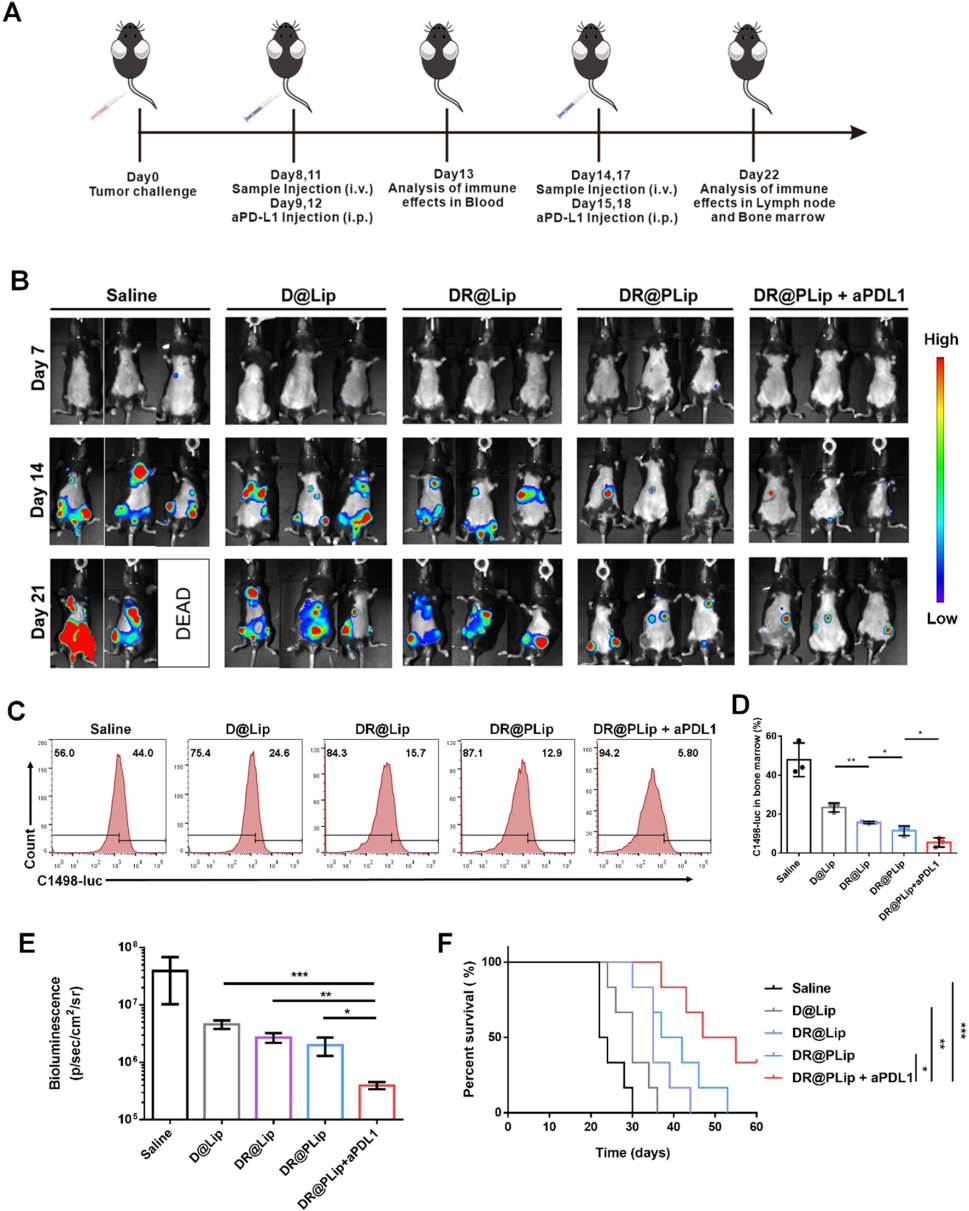
Anti-tumor efficacy in AML model. **A** Establishment of the AML model and treatment scheme. **B** Bioluminescence images of the mice with different treatments. **C** Flow cytometry analysis of the number of C1498 cells in bone marrow (*n* = 3). **D** Statistical analysis of (**C**). **E** Bioluminescence intensity of treated mice on day 22 (*n* = 3). **F** Survival curves of mice (*n* = 6). Data are shown as mean ± SD. *P* values were calculated by Student's t-test (**D**, **E**) and log-rank (Mantel-Cox) test (F), *p < 0.05, **p < 0.01, ***p < 0.001

The C1498-luc model was constructed in C57BL/6 mice for the evaluation of the therapeutic effects in vivo (Fig. 4A). The intensity of bioluminescence was related to the therapeutic effect. As shown in Fig. 4B–E, the bioluminescence signal of DR@PLip + aPD-L1 is the weakest, reflecting the best therapeutic effect. First of all, the PM coating prolonged the circulation time of DR@PLip and promoted C1498 cells targeting in blood. Secondly, Rg3 had a certain synergistic effect on DOX to stimulate ICD effect sufficiently, which was beneficial to the antigen presentation of DCs and the activation of effector T cells. Finally, effector T cells combined with immune checkpoint suppression exerted a strong immunotherapy effect. Consistently, mice received the DR@PLip + aPDL1 treatment showed the lowest numbers of C1498-luc cells compared with other groups. As for the survival curve (Fig. 4F), mice in the DR@PLip + aPD-L1 group showed the longest survival period, and 80% of the mice survived by day 60, which was consistent with the treatment effect. Moreover, due to the activation of immune system, effector T cells could penetrate deeply into the bone marrow and kill the residual tumor cells, which showed superiority against tumor recurrence.

### Immune activation in vivo

**Fig. 5:**
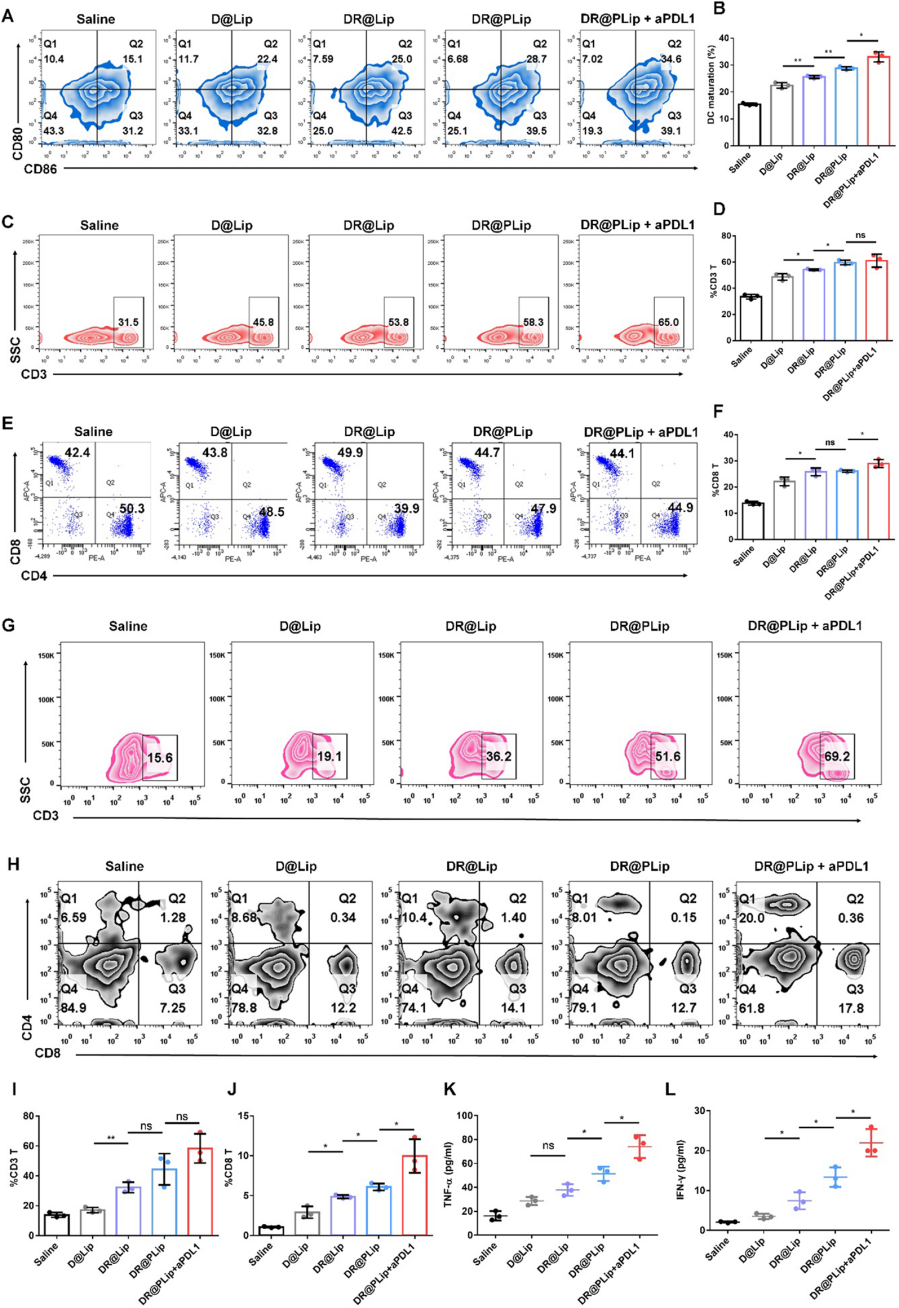
Immuno-activation in vivo. **A** DC maturation (CD80^+^CD86^+^ of CD11c^+^ BMDCs) in the lymph nodes detected by flow cytometry. **B** Statistical analysis of (**A**) (*n* = 3). **C** Proportion of peripheral CD3^+^ T cells on day 8. **D** Statistical analysis of (**C**) (*n* = 3). **E** Proportion of CD8^+^ T cells and CD4^+^ T cells (gated on CD3^+^ T cells) on day 8. **F** Statistical analysis of proportion of peripheral CD8^+^ T cells (*n* = 3). **G** Proportion of CD3^+^ T cells in bone marrow. **H** CD8^+^ T cells and CD4^+^ T cells (gated on CD3^+^ T cells) on day 22. **I** Statistical analysis of (**G**) (*n* = 3). **J** Statistical analysis of proportion of CD8^+^ T cells in bone marrow (*n* = 3). **K** TNF-*α* and (**L**) IFN-*γ* in serum on day 8 detected by ELISA (*n* = 3). Data are shown as mean ± SD. *P* values were calculated by Student’s t-test, *p < 0.05, **p < 0.01, ns means no significant difference

It was shown that the co-delivery of Rg3 and DOX significantly enhanced the tumor ICD, thus promoting the DC maturation and T cells activation to provoke effective immune response. Therefore, the DC maturation in lymph nodes was detected by flow cytometry. As shown in Fig. 5A & B, the proportion of CD80^+^CD86^+^ DCs in the DR@Lip group reached 25.0%, which was significantly higher than that of the D@Lip group due to the enhanced ICD. Then blood samples were collected for T cell analysis. As shown in Fig. 5C–F, the proportion of CD3^+^ T and CD8^+^ T cells in the DR@Lip group (26.1%) was significantly higher than that of the D@Lip group (20.1%) and saline group (13.4%). In addition, aPD-L1 prevented programmed death of T cells and further increase the proportion of CD3^+^ T and CD8^+^ T in the DR@PLip + aPD-L1 group (28.7%). Moreover, TNF-*α* and IFN-*γ* secreted by activated immune cells also increased by 4.7-fold and 6.2-fold respectively in the DR@PLip + aPD-L1 group versus the saline group (Fig. 5K & L). Besides, T cells in bone marrow were be analyzed. As shown in Fig. 5G–J, the proportion of CD3^+^ T (69.2%) and CD8^+^ T (13.84%) cells in the DR@PLip + aPD-L1 group were significantly improved compared with other groups, reflecting the deep penetration into the bone marrow of T cells.

The proportion of T_EM_ cells (CD8^+^ CD44^+^ CD62^−^ T cells) in DR@PLip + aPDL1 group was significantly up to 24.3% (Additional file 1: Figure S8), indicating the formation of long-term immune memory, which was correlated with the prevention of tumor recurrence.

### Biosafety evaluation in vivo

The biosafety of nanosystem is nonnegligible in its in vivo application. As shown in Additional file 1: Figure S9, there was no obvious pathological change in the DR@PLip + aPDL1 group compared with the saline group, indicating the safety and biocompatibility of the nanosystem.

## Conclusion

Traditional chemotherapy showed limited therapeutic efficacy on AML due to its special pathogenesis and the multi-distributed foci [36]. Generally, chemotherapeutic drugs or nanoparticles are hard to penetrate into the bone marrow, which provides the possibility of "resurrection" for the tumor cells that hide in the bone marrow, resulting in high recurrence rate in the clinical treatment of AML [28, 37, 38].

Accordingly, a PM coating DOX/Rg3 co-loaded biomimetic nanosystem (DR@PLip) was explored to achieve “point-to-surface” attack toward AML. In general, the PM coating effectively prolonged the circulation time of DR@PLip thus creating favorable condition for its function. Besides, the natural adhesion of PM to AML cells made it effectively capture AML cells in the blood. Meanwhile, the combination of Rg3 and Dox significantly enhanced the ICD effect and ignited the immunity system. The further combination of aPD-L1 promoted the immune warfare to spread to the whole body, and even deeply attacked the AML cells in the bone marrow. The designed nanosystem (DR@PLip) could not only assist chemotherapy drugs to achieve a powerful blow to the enemy (AML cells), but also further arm the immune system to achieve a comprehensive attack on AML that has wide range of disease areas and deep micro-residue foci. It provided promising strategy against tumor recurrence in clinical treatment of AML.

## Supplementary Information

**Additional file 1:** **Figure S1.** Size distribution and PDI changes of DR@PLip. **Figure S2.** Statistical analysis of cellular uptake of DR@Lip and DR@PLip. **Figure S3.*** In vitro* drug release of free DOX and DR@PLip. **Figure S4.** *In vitro *Rg3 release from DR@PLip. **Figure S5.** Cytotoxicity of R@Lip in C1498 cell line. **Figure S6.** Plasma concentration-time curves of DR@Lip and DR@PLip. **Figure S7.** Statistical analysis of T_1/2_ and AUC_0-t_ of DOX in rats after *i.v.* with DR@Lip and DR@PLip. **Figure S8.** The proportions of T_EM_ cells (CD3^+^, CD8^+^, CD44^+^, CD62L^-^) in spleen. **Figure S9.** H&E staining of the major organs collected from the saline group and DR@PLip+aPDL1 group. **Table S1.** Pharmacokinetic parameters of DOX in rats after *i.v.* with DR@Lip and DR@PLip.

## Data Availability

All data needed to evaluate the conclusions in the paper are present in the paper and/or the Supplementary Materials. Additional data related to this paper may be requested from the authors.
