# Supplementary material for: Biomimetic doxorubicin/ginsenoside co-loading nanosystem for chemoimmunotherapy of acute myeloid leukemia
Source: J Nanobiotechnology. 2022 Jun 14;20:273. doi: 10.1186/s12951-022-01491-w (PMC9195256; doi:10.1186/s12951-022-01491-w)
Supplement: Supplementary file 1 — Additional file 1: Figure S1. Size distribution and PDI changes of DR@PLip. Figure S2. Statistical analysis of cellular uptake of DR@Lip and DR@PLip. Figure S3. In vitro drug release of free DOX and DR@PLip. Figure S4. In vitro Rg3 release from DR@PLip. Figure S5. Cytotoxicity of R@Lip in C1498 cell line. Figure S6. Plasma concentration-time curves of DR@Lip and DR@PLip. Figure S7. Statistical analysis of T1/2 and AUC0-t of DOX in rats after i.v. with DR@Lip and DR@PLip. Figure S8. The proportions of TEM cells (CD3+, CD8+, CD44+, CD62L-) in spleen. Figure S9. H&E staining of the major organs collected from the saline group and DR@PLip+aPDL1 group. Table S1. Pharmacokinetic parameters of DOX in rats after i.v. with DR@Lip and DR@PLip. [file 12951_2022_1491_MOESM1_ESM.docx]

**Additional file 1**

Biomimetic doxorubicin/ginsenoside co-loading nanosystem for chemoimmunotherapy of acute myeloid leukemia

Mo Chen^a^, Yingyu Qiao^a^, Jie Cao^a^, La Ta^a^, Tianyuan Ci^b,^*, Xue Ke^a,^*

^a^Department of Pharmaceutics, China Pharmaceutical University, Nanjing, Jiangsu province, 210009, China

^b^Department of Pharmaceutical Sciences, Shanghai University of Traditional Chinese Medicine, Shanghai, 201203, China

Corresponding author:

Corresponding authors: Xue Ke*

Mailing address: China Pharmaceutical University, Xuanwumen Campus, No.24, Tongjiaxiang, Gulou District, Nanjing, 210009, China

E-mail: [kexue1973@vip.sina.com](mailto:kexue1973@vip.sina.com)

Corresponding authors: Tianyuan Ci*

Mailing address: Shanghai University of Traditional Chinese Medicine, Shanghai, 201203, China

Tel: +86-21-51322197

E-mail: [citianyuan_cpu@163.com](mailto:citianyuan_cpu@163.com)

**This word file includes:**

Fig. S1. Size distribution and PDI changes of DR@PLip

Fig. S2. Statistical analysis of cellular uptake of DR@Lip and DR@PLip.

Fig. S3. *In vitro* drug release of free DOX and DR@PLip.

Fig. S4. *In vitro* Rg3 release from DR@PLip.

Fig. S5. Cytotoxicity of R@Lip in C1498 cell line.

Fig. S6. Plasma concentration-time curves of DR@Lip and DR@PLip.

Fig. S7. Statistical analysis of T_1/2_ and AUC_0-t_ of DOX in rats after *i.v.* with DR@Lip and DR@PLip.

Fig. S8. The proportions of T_EM_ cells (CD3^+^, CD8^+^, CD44^+^, CD62L^-^) in spleen.

Fig. S9. H&E staining of the major organs collected from the saline group and DR@PLip+aPDL1 group.

Table S1. Pharmacokinetic parameters of DOX in rats after *i.v.* with DR@Lip and DR@PLip.


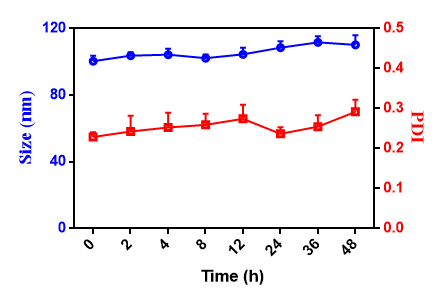


Fig. S1. Size distribution and PDI changes of DR@PLip in 48 h at DMEM medium + 10% FBS (*n* = 3).

Fig. S2. Statistical analysis of cellular uptake of DR@Lip and DR@PLip. *P* values were calculated by Student's t test, ****p* < 0.001.


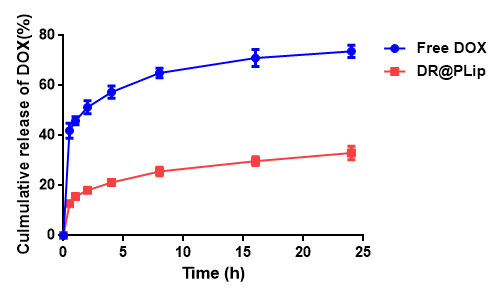


Fig. S3. *In vitro* drug release of free DOX and DR@PLip. Results are expressed as mean ± SD (*n* = 3).

Fig. S4. *In vitro* Rg3 release from DR@PLip. Results are expressed as mean ± SD (*n* = 3).

Fig. S5. Cytotoxicity of R@Lip in C1498 cell line (*n* = 3).

Fig. S6. Plasma concentration-time curves of DR@Lip and DR@PLip after intravenous injection in rats (*n* = 3).


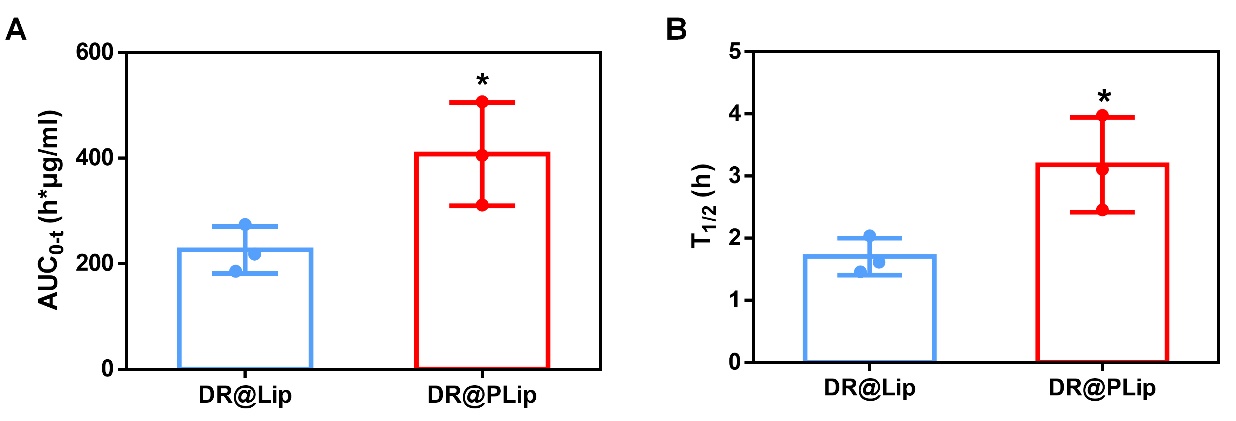


Fig. S7. Statistical analysis of (A) T_1/2_ and (B) AUC_0-t_ of DOX in rats after i.v. with DR@Lip and DR@PLip. *P* values were calculated by Student's t test, **p* < 0.05.


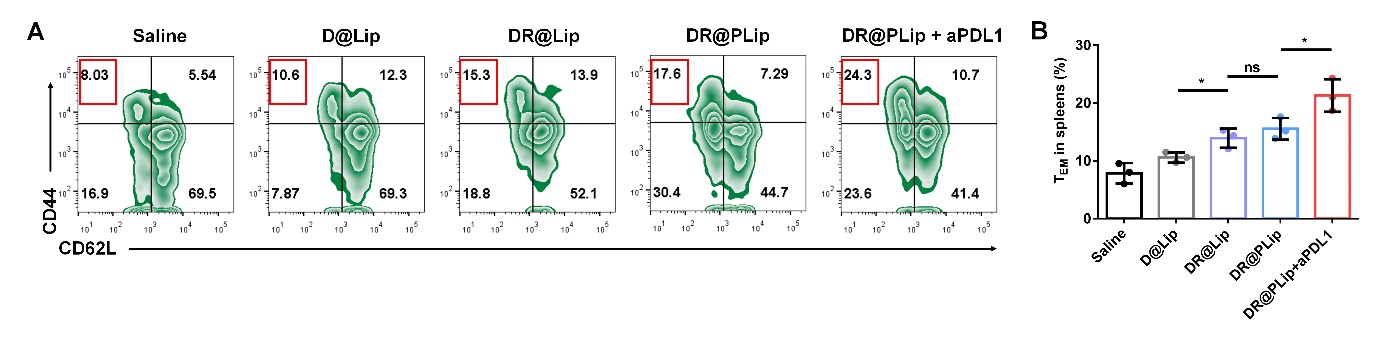


Fig. S8. (A) The proportion of T_EM_ cells (CD3^+^, CD8^+^, CD44^+^, CD62L^-^) in spleen (*n* = 3). (B) Statistical analysis of (A). *P* values were calculated by Student's t test, **p* < 0.05, ns means no significant difference.


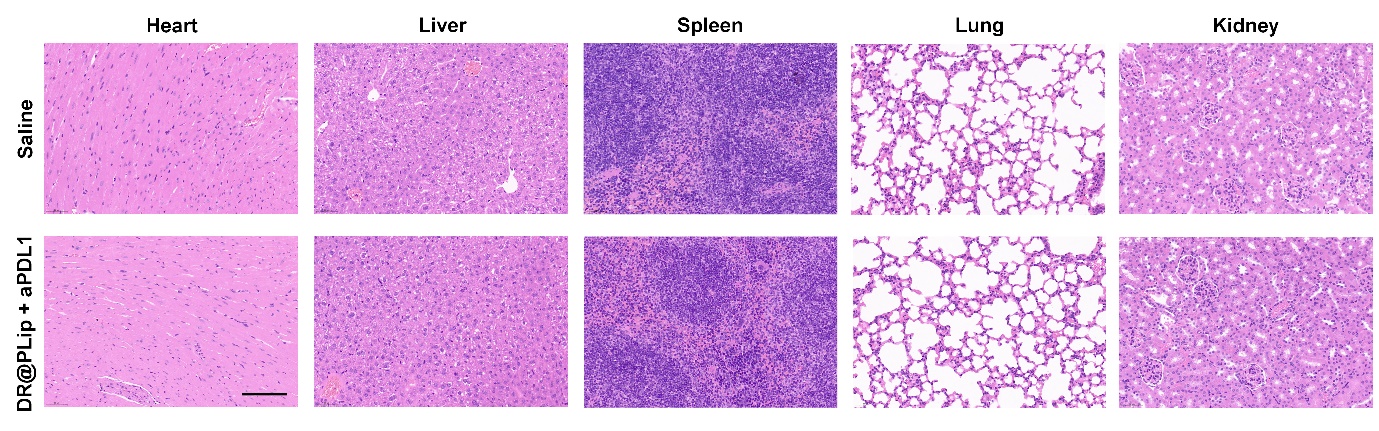


Fig. S9. H&E staining of the major organs collected from the saline group and DR@PLip+aPDL1 group (scale bar, 100 μm).

Table S1 Pharmacokinetic parameters of DOX in rats after *i.v.* with DR@Lip and DR@PLip at the dose of 5 mg kg^-1^ DOX.

| Parameter | DR@Lip | DR@PLip |
| --- | --- | --- |
| C_max_ (μg/ml) | 94.8 ± 3.65 | 95.74 ± 3.16 |
| T_1/2_ (h) | 1.70 ± 0.30 | 3.18 ± 0.76 |
| AUC_0-t_ (h*μg/ml) | 226.02 ± 44.78 | 407.42 ± 97.78 |
| CL (L (h kg^-1^)^-1^) | 4.54 ± 0.87 | 2.55 ± 0.062 |
